# Virtual Reality in Health Professions Education: Qualitative Descriptive Study of Educators’ Perspectives

**DOI:** 10.2196/52925

**Published:** 2026-06-26

**Authors:** Tak Wing Yu, Michael Rowe, Jose Frantz

**Affiliations:** 1 Department of Physiotherapy, University of the Western Cape, Community and Health Science Building, Bellville Campus, 14 Blanckenberg Street, Bellville, Cape Town, Western Cape, 7530, South Africa, +2782 314 8525; 2 Digital Innovation in Health & Social Care, School of Health & Care Sciences, University of Lincoln, Lincoln, United Kingdom; 3 University of the Western Cape, Physiotherapy, University of the Western Cape, Bellville, South Africa

**Keywords:** virtual reality, VR, teaching and learning, educators, teachers, health professions education, medical education, technology education, technology-enhanced learning, TEL, education, experiences, attitudes, perspectives, immersive

## Abstract

**Background:**

As virtual reality (VR) technology has become more accessible, the potential of this technology has been increasingly investigated in higher education institutions to improve teaching and learning. While VR can provide immersive experiences to support visualization and active learning, its adoption in health professions education is often limited by high costs, technical complexity, and a lack of pedagogical fit. Furthermore, the educator, who is critical to curriculum design, is underrepresented in the discourse on VR integration, especially in resource-constrained environments in South Africa.

**Objective:**

The purpose of this study was to investigate the perceptions of health professions educators regarding the potential value of immersive VR in the educational process, the implementation barriers, and the requirements for successful curriculum integration at a South African university.

**Methods:**

A qualitative exploratory descriptive design was used. Eighteen educators (N=18) from a wide variety of health disciplines (including physiotherapy, occupational therapy, nursing, and dentistry) were recruited from the Faculty of Community and Health Sciences. Participants interacted with 3 immersive VR applications (The Body VR, Sharecare You VR, and Wraith VR), which were chosen to represent varying levels of clinical complexity. Semistructured interviews were used to explore their perceptions. Data were analyzed using reflexive thematic analysis in accordance with the framework of Braun and Clarke. Sample size adequacy was determined using information power.

**Results:**

Five overall themes were constructed from the data: (1) the experience of VR, where educators enjoyed the engagement but initially felt that VR was a “gaming” novelty; (2) teaching and learning preferences, where VR was considered a potential tool for authentic and individualized learning; (3) challenges of VR, where issues of visual overload, cybersickness, and logistical barriers were reported; (4) clinical competency and patient safety, where VR was valued as a safe space to manage errors; and (5) curriculum integration, where there was a preference for scaffolding the use of VR from foundational anatomy in the junior years to procedural simulation.

**Conclusions:**

Educators have a favorable view of VR as a powerful supplemental tool that can help boost student engagement and bridge the gap between theory and clinical practice. However, successful implementation involves more than purchasing hardware; it requires a strategic “scaffolded integration” approach that ensures VR applications align with specific curricular outcomes. It is the role of institutions to not only provide technical assistance but also pedagogical training and support for infrastructure to transform a technological gimmick into an ongoing educational resource.

## Introduction

### Background

Virtual reality (VR) technology is no longer restricted to the gaming industry; it has entered the classroom as a pedagogical tool in health professions education (HPE) [1]. This step has provided learners with an immersive environment that can facilitate visualization of complex anatomical structures, which cannot be easily created physically without causing patient harm [2]. Extensive evidence exists that VR is useful in facilitating learning, spatial awareness, and motivation [3-6]. However, as the hype around the technology wears off, the question has moved past whether it will work to how we keep it going. This is particularly vital in resource-constrained settings, where implementing new technology can be a challenge amid other priorities within the education system [7].

Even though VR was initially promoted as a low-cost alternative to high-fidelity simulation-based learning (SBL) techniques that require expensive mannequins, consumables, and separate rooms, it does not replace any training tool. The finances are delicate [8-11]. VR involves a high initial cost for equipment, software, and technical support [12]. According to Ravichandran and Mahapatra [12], the initial expenses are high, but VR can be scaled in the long run; once the app is purchased, many thousands of students can use it at a very minimal incremental cost compared to the current expenses of cadaveric dissection or wet laboratories.

Although VR has been cited as having advantages over other pedagogical techniques, including SBL, it also presents realistic challenges in HPE. Two recent studies conducted by Nemani [13] and Soltani and Rostami [14] emphasize that successful integration is often hindered not only by hardware limitations and constraints but also by institutional and organizational constraints, including people (human resources). Furthermore, the exploration of “educator readiness” is required. Educators are the key gatekeepers of the curriculum. If they view VR as a “gimmick” or lack the pedagogical competency to integrate the technology, there is a risk of marginalizing it as a supplementary activity rather than a core learning modality.

This challenge is more pronounced in developing countries such as South Africa, where, due to the digital divide and infrastructural constraints, the use of immersive technologies can be a daunting prospect [15]. Mondal and Mondal [16] noted that to prevent the waste of technology, a detailed alignment with the local curriculum is required. However, educators’ voices on the use of VR are underrepresented in the global literature and are predominantly biased toward high-income nations [17]. Understanding how educators in South Africa view the pedagogical value, ease of use, and logistical overhead of VR is imperative for developing implementation strategies that are culturally and institutionally sustainable.

Therefore, the purpose of this study was to explore the perceptions of health professions educators at a South African university regarding the immersion of VR. Furthermore, the identification of potential pedagogical challenges (such as implementation) and possible solutions and strategies was explored.

### Purpose of the Study

Health professions educators are among the key stakeholders in incorporating VR into the curriculum. Therefore, exploring educators’ perspectives on the use of VR is fundamental to the sustainable integration of VR in HPE. Thus, this study seeks to understand educators’ perceptions of VR in HPE, with special reference to perceived educational value, implementation difficulties, and issues in curriculum integration.

## Methods

### Study Design and Setting

This study used a qualitative, exploratory, and descriptive design within an interpretivist paradigm. This approach was chosen to bring out participants’ lived experiences as health professions educators as they interacted with novel VR technologies, thereby allowing for a rich, context-dependent understanding of their pedagogical perceptions [18,19]. The study was conducted in the Faculty of Community and Health Sciences at one of the research-intensive universities in the Western Cape, South Africa. It was an interdisciplinary setting that encompassed physiotherapy, occupational therapy, nursing, psychology, and dentistry, among other educational areas within the health sciences.

### Participant Selection and Recruitment

Academic employees were selected through purposive sampling to ensure that they were actively engaged in teaching at the Faculty of Community and Health Sciences. Advertisements for recruitment were sent via email to the faculty and distributed at department meetings. To secure a wide range of opinions, maximum variation methods were used to select participants from various disciplines with different degrees of teaching experience (junior lecturers, lecturers, senior lecturers, and associate professors) and different levels of technology experience (junior or experienced). Participant recruitment continued until a certain level of information power was achieved [3]. The continuous recruitment ensured adequate specificity and depth to achieve the study’s goals and objectives, and 18 participants were recruited.

### VR Intervention and Procedure

All participants were taken through a structured VR workshop prior to the interviews to establish a baseline of experiential knowledge. The lessons were conducted in a special simulation laboratory with HTC VIVE Cosmos VR goggles. The participants were requested to experience 3 particular VR applications, which were selected to illustrate different pedagogical affordances:

1. The Body VR: a narrative storytelling journey that takes the user within the bloodstream, via an immersive approach, throughout the body. The application is designed to illustrate abstract physiological concepts (key characteristics and components: passive and observational).
2. Sharecare You VR: a highly interactive anatomical atlas allowing for the dissection and manipulation of organs (key characteristics and components: active and exploratory).
3. Wraith VR: a procedural simulation requiring manual dexterity and sequence recall (key characteristics and components: active and procedural).

Each session lasted about 20 to 30 minutes, and participants were given time to familiarize themselves with the hardware and interface before reflecting on the experience.

More details on the VR application are provided in Multimedia Appendix 1.

### Data Collection

Data were collected via semistructured individual interviews immediately after VR exposure (Multimedia Appendix 2). The semistructured interview guide was developed using the CAMIL (Cognitive Affective Model of Immersive Learning) framework [20]. Makransky and Petersen [20] framework provides a grounded basis for identifying key constructs relevant to the learning context, including interest, motivation, embodiment, and self-regulation. Although the thematic analysis was conducted using the reflexive thematic analysis (RTA) inductive approach, without using CAMIL as an a priori coding framework, it allowed themes and subthemes to emerge from the interviews rather than from existing theory and literature. The primary focus of the interviews revolved around the following:

1. Pedagogical affordances: What are the key components you think are needed for a successful VR integration in the educational system?
2. Implementation barriers: What is hindering the ease of using VR?
3. Curricular alignment: In which module will you implement them (describe how the module is able to link to VR)?

Interviews were audio-recorded with participants’ consent. The primary researcher wrote field notes and reflexive memos throughout the data collection process to document nonverbal cues and early analytical thoughts.

### Data Analysis

#### Thematic Analysis

The data analyzed utilized the RTA approach, as outlined by Braun and Clarke [21]. This was an iterative 6-phase process that involved the following:

1. Familiarization: repetitive listening to audio recordings and reading transcripts in order to immerse oneself in the data.
2. Coding: sequential production of systematic semantic codes and latent codes for the entire dataset.
3. Generating themes: pulling together codes into possible themes that represent shared patterns of meaning.
4. Reviewing themes: reviewing themes against the coded extracts and the overall dataset to ensure that they create a coherent narrative.
5. Defining and naming themes: developing the details of each theme to make them clear and distinct.
6. Writing up: writing the final report.

#### The Role of Artificial Intelligence in Data Analysis

After the primary researcher completed the initial analysis, the artificial intelligence (AI) model was provided with the human-developed codebook and the anonymous semistructured interview transcript for analysis. The initial prompt involved giving the AI model a role; the research aim, objective, and research question were provided (eg, “You’re a Professor at a University working in the Physiotherapy Department”). The AI model was instructed to identify excerpts that differed from or codes that contradicted the researchers’ primary findings. The results from the AI model were treated as unverified notes, and gaps were identified; they were cross-referenced by the research team against the raw audio and transcript to ensure contextual accuracy and truthfulness. The final decision on the results was determined by the researchers.

### Trustworthiness and Rigor

Rigor was established by using the criteria of credibility, transferability, dependability, and confirmability [22].

- Credibility: ensured by spending time with the data and researcher reflexivity. The researchers scheduled quarterly meetings with an institutional researcher to discuss, debrief, and provide feedback and questions about the study’s analysis process.
- Transferability: enhanced through the provision of detailed descriptions of the study setting, participants, and the VR intervention to enable the reader to judge whether they might apply to other contexts.
- Dependability and confirmability: an audit trail was maintained for key methodological decisions, including the development of a codebook, subthemes, and themes.

### Reflexive Statement

The lead researcher is a physiotherapy lecturer working in the same faculty as all participants, with an understanding of the educational environment and potential biases. A desire to deploy VR in health sciences education, driven by a disciplinary background as a physiotherapist, was recognized at the outset as likely to affect coding and interpretation processes. This was moderated through reflexive memos during data collection and analysis processes to expose and challenge underlying biases. Regular supervisors’ meetings helped to externalize thinking. Participants were encouraged to know that both critical and positive responses were sought and valued. During the interviews, participants were reminded of the lead researcher’s role. A semistructured interview was used to allow participants to elaborate on their experiences and perceptions rather than simply responding to an investigative prompt.

### Ethical Considerations

Ethics approval was granted by the Humanities and Social Sciences Research Ethics Committee of the University of the Western Cape (reference HS22/6/55). An information sheet and a consent form were sent prior to the interviews, and the participants signed the consent form before starting the interview process. All identifiable information was removed from the transcript before data analysis. To protect data privacy during AI processing, only deidentified textual data were input into ChatGPT. Participants in this study did not receive any stipend or compensation.

## Results

### Demographics and Background

The participants’ ages ranged from 32 to 54 years, with a mean age of 42 (SD 9.44) years. Occupational therapy, physiotherapy, dentistry, nursing, psychology, social work, sport, recreation and exercise science, biokinetics, and the Interprofessional Education Unit were represented. Details of the participants are available in Multimedia Appendix 3.

### Themes, Subthemes, and Codes

The analytical process involved the creation of 105 initial codes, inductively developed from the interview data and field notes (Multimedia Appendix 4). These codes represented fragmented ideas, experiences, and perceptions expressed by participants and remained data-driven during initial familiarization and the initial round of coding. Through an iterative and reflexive process, the codes were compared, sorted, and refined to highlight patterns of meaning. Similar codes were then organized into 16 subthemes that captured common pedagogical, experiential, and contextual issues from participants’ experiences. These subthemes were grouped into 5 high-level themes that captured higher-level patterns of meaning related to educators’ views on VR in HPE. Themes were developed based on analytic consistency and interpretative meaning rather than on counts, as is typically the case in RTA. Consultation between the authors on codes, subthemes, and themes was ongoing throughout the data analysis to ensure the representativeness and fit of the codes and themes. This also enabled them to be defined and redefined through data analysis.

### The Educator’s Experience in the Use of VR

Before this study, most participants had only heard of or had a surface-level understanding of VR terminology. Some participants had VR experiences related to watching media or videos on their phones. Participant 14, a dentist, said, “Nothing was done before, not even a VR video.” This sentiment was echoed by participant 3, a physiotherapist, who noted the transition from gaming to education: “Gaming, I’ve mostly seen it in gaming and gradually… [they are] trying to bring it into the teaching environment as well.” For many of these professionals, the research project provided their first physical encounter with fully immersive virtual worlds. It is important to note that the interview questions addressed VR in general, but the participants’ responses were heavily influenced by the high-fidelity, fully immersive experiences provided during the study.

The emotional reaction to this was largely positive, even though there was no prior experience, and it was defined by an emotional wow factor. The most frequently used words by the participants to describe their participation were immersion, excitement, and fun. Indicatively, a psychologist, participant 5, reported feeling deeply emotional about the experience of cellular visualization: “I felt like I was inside the cell. I was struck. I adjusted the wow factor to that. Wow. What is it? I have never experienced anything of the kind; therefore, in my case, it was the first time that I was exposed to the virtual environment.” This feeling of belonging to the setting is a characteristic feature of high-presence VR and represents a major change from traditional classroom observation. Participant 3 (physio) pointed out the direct influence of these affective factors in the learning process: “It feels more fun, more fun. It is more immersive, and as I listen to the models, I can see their labels and look around to see where these models or structures fit.” This participant also mentioned that this would be more interactive for students; it is more fun, suggesting that the fun of the VR experience might be an effective motivator for student engagement.

### Teaching and Learning Preferences

The participants found VR to be a powerful alternative modality that can add value to education through its immersive, interactive nature. Instead of being a passive visual representation, the technology was viewed as a stimulus for various learning orientations, such as exploratory, active, and individualized learning.

### Immersive Learning

The ability to immerse oneself in the content deeply, the feeling of being physically present, was considered to be one of the main motivators of retention. Participant 7 (sport science) claimed that the value of VR lies in its ability to aid memory through active participation: “The one value is that it helps you to remember. As you are part of what you are learning.” This experiential “being part of” the content facilitates deep encoding and retrieval, as the student is not just observing a process but living it. Participant 9 (sport science) emphasized the importance of spatial perspective in developing clinical insight: “By seeing what happened from a difficult angle, it can help you understand [the condition] after a client has had surgery.” This ability to manipulate viewpoints and observe clinical conditions from angles inaccessible in conventional environments provides a deeper level of understanding.

### Active and Individualized Learning

Educators stressed the transition from passive receiving to active agency. Participant 7 (ERES) noted the importance of student autonomy: “The fact that you can choose your options, you can go back and choose which part of the body you can do the tests, see where the breakages are, how things are changing and flowing. That makes you feel like you’re part of the lesson that’s happening, and not just someone who’s receiving the old ways of teaching.” This sense of participation shifts the student’s role from a consumer of information to an active investigator. Participant 15 (dentist) reflected on their own learning style and how VR might have aided their studies: “The type of student I was, and I am currently seeking help with my studying. If I can visualize it, it stays in my memory, as opposed to me going to a lecture, listening and then going home.” This highlights the technology’s ability to cater to those who struggle with traditional lecture-based formats. Participant 8 (ERES) observed that “It gives the opportunity for the student who learns slightly slower. I’m thinking of individualizing learning. It is almost custom-made for each student. It’s like apps on your phone. I could decide how fast we are moving.” This customization addresses the diverse needs of learners and enables a more inclusive educational experience, much as specialized programs can help diverse groups express themselves creatively and overcome repressed emotions.

### Authentic Learning

Lastly, the participants discussed the possibility of simulating near-real clinical situations. Participant 14 (dentist) stated, “Ultimately, we would like to strive for authentic learning experiences, and VR could offer that based on the setup scenario.” This can be achieved through VR, which serves as a safe, high-fidelity simulation of a clinical setting or a virtual human body.

### Clinical Competencies and Patient Safety

One recurring issue among educators was the risk of student errors in the real-world clinical environment. The participants often placed VR as a necessary, nonthreatening intermediate through which it is possible to experience safe failure. Participant 7 (sport science) found value in the nonthreatening nature of the technology: “I could make mistakes. But I felt it was nonthreatening, which is definitely a good thing.” Participant 6 (social work) pointed to the moral necessity: “Even if the students make a mistake...they realize they learn from making mistakes. However, in the real environment, it is not possible, as you realize the patient might die.” This concept is transformative for clinical training, as it allows students to learn from mistakes that would be unacceptable in a real-world setting.

Students with this safety affordance were directly associated with preclinical preparation and the number of hours of clinical experience. Participant 2 (physio) stated, “VR will improve the student’s confidence. More so, to promote and enhance their experiences during clinical placements. Because students would develop competence prior to working with real patients.” This is supported by the fact that VR can accelerate skill acquisition and enhance overall clinical outcomes. Educators also regarded VR as a means of overcoming institutional barriers, including limited access to hospitals. Participant 8 (sport science) observed that VR could be used when students “cannot go into hospital settings,” as it can “bring the clinical environment to the students” and expose them to complex equipment: “You’re placing them in a [virtual] hospital room where they can see all the valves or machines we use in physio; whatever it is, they’ll be able to see it.” Participant 12 (physio) even proposed a formal recognition of virtual training: “So you could allocate clinical hours as well, not only when they are on block to prepare but also to give them that clinical experience and confidence.” Participant 10 (sport science) suggested that “the students’ confidence and also the patient [functional] outcome increases, maybe it will even increase students’ [clinical] competence.” This highlights a shift toward accepting virtual simulation as a legitimate part of professional clinical hours.

### VR and Curriculum Alignment

Participants stressed that VR’s effectiveness depends on its scaffolded integration into the curriculum rather than its use as a generic add-on. Discipline-specific alignment, in which content aligns with the anticipated outcomes of a particular module, was very popular. For example, the participants proposed that anatomical applications are most appropriate in junior years (first and second years), whereas procedural simulations (eg, Wraith VR) should be used with senior students (third and fourth years) or postgraduate students. Participant 10 (sports science) observed that VR might familiarize junior students before they are expected to perform complex tasks, including the first surgical extraction.

Participants’ opinions on the level of integration were inconclusive. Some, such as participants 6 (social work) and 8 (ERE), said they would like VR to become a primary teaching tool, possibly replacing traditional lectures. Others, such as participant 3 (physio), noted that “Theory and textbooks, they do have their place. And I think VR can supplement.” However, participant 10 (sport science) suggested an “equal” balance: “I think it will be equal. I would use that equal...I would send them the theoretical information before the time. Then, the main tool is to put it into practice, because that is my work, applying it.” This variety of opinions suggests that the optimal level of integration may vary across disciplines and specific learning objectives. Irrespective of the level of use, teachers such as participant 8 (ERES) stated that “the technology should be linked to an outcome module,” and that, without understanding the pedagogical fit, the implementation process will be unsuccessful.

### Challenges of VR Implementation

The enthusiasm notwithstanding, a number of challenges at different levels were identified, the main ones being cognitive load, cybersickness, technical, logistical, and financial obstacles.

The first problem identified was the risk of visual and mental overload. While powerful visualizations are a strength of VR, they can also be overwhelming for those not accustomed to the stimulus. Participant 11 (ERES) cautioned, “It might be too overwhelming with the visual and hearing all information, all at once, for initial exposure might be a little bit too much if that’s their first exposure to content.” Participant 6 (social work) noted that this stimulus is not suitable for everyone: “This would be beneficial for those who need this kind of [visual] stimulus, and then you have the other side of the spectrum where it would be overwhelming for those who do not.”

There were also complaints of physical discomfort and usability issues. Physical discomfort, specifically “cybersickness,” was reported. Participant 4 (psychologist) described a loss of balance: “I lost a bit of balance. And my knees suddenly became weak.” Participant 2 (physio) experienced visual issues: “At times, it got a bit blurry and then cleared up. And there were lags.” These physiological responses are common in early-stage VR adoption and highlight the need for sessions of appropriate duration and high-quality hardware to minimize vestibular conflict.

Technical obstacles, including the difficulty of controller buttons, served as a major distraction from the learning objectives. Participant 4 (psychologist) noted that “The buttons on the controller were difficult to use; it was complicated.” Participant 14 (dentist) found that the interface interfered with the learning objectives: “The biggest distraction for me is that if I pressed the button, I was either too high or too low on the wrong side.” Participant 2 (physio) also observed that “managing the material, my hands with the controls took a bit of practice.” These findings suggest that the user interface must be intuitive to prevent the technology itself from becoming a barrier to learning.

Lastly, institutional obstacles such as cost and scalability were raised. Participant 6 (social work) questioned the university’s ability to invest: “We’re talking about thousands of rands. I don’t know if the university has money to invest.” The high cost of headsets and software licenses makes scalability difficult. Participant 4 (psychologist) pointed out that “We need to have more than one, or five for the number of students that we have, for it to be valuable.”

Class size is a major logistical hurdle. Participants 3 (physio) and 15 (dentist) asked “A class of 75‐80 students, how long will it take them to rotate one machine?” This rotation time can be a significant drain on teaching resources and limit the time each student can spend in the virtual environment. For VR to be truly effective in a large-class setting, multiple devices and efficient scheduling are required. The challenge of managing limited resources while providing a high-quality experience is a common theme in fields undergoing professional development.

## Discussion

### Principal Findings

Our study asked health professions educators for their perceptions after using immersive VR to teach certain courses or modules. The study found that educators perceive VR as an educational tool that motivates students, helps them understand complex concepts, and encourages active learning. Other studies have also found that VR is more motivating and can help students understand spaces and engage in active learning [3-6].

Participants in this study indicated that VR will be useful only when aligned with the course and pedagogy. Participants suggested that VR should be employed as part of a lesson aligned with the Course Intended Learning Outcome, rather than simply for “using” technology in teaching. This approach reinforces the importance of alignment and provides the educator and students with a clear aim and learning goal. As with any technology implementation, the initial attractiveness and novelty will maintain students’ attention; however, for long-term educational value, sustainability and support are required [23]. These supports are provided by different levels of the institution. For example, a lab technician (manpower) and storage space (infrastructure) are required [24].

Participants, as educators, highlighted that VR is useful for learning from mistakes without risk to the patient, thus creating a safe environment for students to explore and be unsuccessful without causing actual harm to the patient. A safe learning environment is important for students, as it helps them construct knowledge from the provided content [25]. Besides a safe learning environment, it is important for students to learn from their mistakes [26]. VR can provide immediate feedback to help students review their mistakes and learn from them. Additionally, VR offers high replayability, enabling students to return to the virtual environment and redo the learning activity until the desired outcome is achieved.

### Comparison With Prior Work

The high expectations individuals have regarding their affective reactions align with earlier research findings, indicating that immersive VR can positively influence engagement and motivation [4,6]. This is in line with larger trends in the adoption of educational technology, in which emerging tools tend to initially produce a surge of excitement before plateauing into more lasting and meaningful applications [27]. However, this paper provides some sense of the impact of novelty by demonstrating that teachers are aware of it and emphasize the necessity of lasting pedagogical value.

Findings can also be interpreted using the CAMIL [20], particularly regarding the constructs of interest, motivation, embodiment, and self-regulation. The descriptions of immersion, interaction, and a deeper understanding of the space provided by participants indicate the embodiment pathway proposed by CAMIL, in which learners experience the virtual environment and think more profoundly. In the meantime, this paper also illuminates important issues such as cognitive load and usability. The respondents reported visual overload and difficulty navigating the VR interface, particularly at the beginning. Even though CAMIL also acknowledges the influence of cognitive load in the context of immersion, these findings suggest that the extraneous cognitive load may also be driven by a lack of experience with the corresponding technology. This confirms the importance of preparatory measures, such as orientation or pretraining, that can enhance usability and reduce mental load [28].

The focus on curriculum alignment and scaffolded integration can be viewed as a valuable addition to the literature. Although the literature shows that VR is effective in enhancing learning outcomes [5,6], very few studies have examined how VR should be integrated into learning programs. The respondents in this research reported a progressive format in which VR implementation must be introduced in basic subjects, that is, anatomy, during the early years, and its application in more complex simulations of procedures during the later stages. This is consistent with competency-based education systems such as the South African National Qualifications Framework (SANQF) [29], which focus on developmental advancement and conformity with professional skills.

The perceived value of VR as a safe environment for practice and error aligns with the SBL literature [30,31]. Nonetheless, teachers in the present research considered VR as a complement, not a replacement, for conventional teaching and simulation techniques. Although VR has its benefits in terms of scalability and accessibility [12,32], its weaknesses include the lack of haptic feedback, which may limit its ability to substitute for hands-on clinical training.

Lastly, the barriers identified in this study, such as cost, infrastructure, and technical complexity, are similar to those reported in past studies [13,16]. However, this study also highlights that these issues are not only technical but also involve institutional readiness and teachers’ abilities, which justifies the need to examine them holistically for implementation.

### Future Directions

The future directions of research in VR are as follows:

1. Future research should focus on longitudinal and experimental studies, as this will provide stronger evidence of VR’s effectiveness relative to traditional and blended learning paradigms. The perceived effect of VR on learning, skills, and patient safety has been determined [33-35].
2. Further research could explore a design principle or a set of instructions for integrating VR into the curriculum, given the importance of aligning the learning outcomes with the VR application.
3. From an implementation standpoint, research should be conducted to explore scalable, cost-effective delivery models for integrating VR, especially in low-resource environments. This may involve alternative VR delivery options, such as desktop or mobile platforms, as well as other infrastructure models.
4. Finally, there is a need for future research on integration at the curriculum level, exploring the methodical integration of VR into HPE curricula and mapping it to competency frameworks. This work is critical to support the progression from VR as an innovative tool to VR as an embedded tool in HPE.

### Limitations

There are several limitations to consider.

1. To begin with, this research was conducted at a single institution and used a small sample (N=18), which may limit the generalizability of the results to other geographical or institutional settings. Nevertheless, the concept of information power, in which sample adequacy is judged by the relevance and richness of the information in relation to the study aim, was used to assess sample adequacy. In this study, the specificity of the educator sample and the thoroughness of the RTA indicated that the data held sufficient information power to support meaningful interpretation. In addition, participants’ years of experience were not collected, even though this could have informed their curriculum and VR-teaching practices.
2. The perceptions were gathered after a brief demonstration; therefore, the responses could also have been influenced by novelty bias rather than by long-term pedagogical usefulness.
3. This research did not measure learning outcomes; although perceptions provide a clear picture of feasibility, further research is needed to quantify objective skills acquisition.
4. Since only 3 applications were shown, the results might not be representative of the overall capabilities of more sophisticated or custom VR systems that are under development.

### Conclusion

To sum up, health professions educators view VR as an engaging and potentially disruptive learning tool when designed structurally in line with educational objectives. Although VR offers distinct benefits in motivation, immersion, and scalability, it should be used as a strategic addition to current teaching and simulation techniques. The key to successful implementation in resource-constrained settings lies in shifting the current trend of momentary enthusiasm toward a systematic examination of curricular constraints and intended evaluation methods. VR has great potential to enhance the quality and safety of HPE by addressing institutional barriers and offering pedagogical support.

## Supplementary material

10.2196/52925 Multimedia Appendix 1 Virtual reality application details.

10.2196/52925 Multimedia Appendix 2 Semistructured interview guide.

10.2196/52925 Multimedia Appendix 3 Faculty of Community and Health Science.

10.2196/52925 Multimedia Appendix 4 Themes, subthemes, and codes.

## Abbreviations

AI: artificial intelligence
CAMIL: Cognitive Affective Model of Immersive Learning
HPE: health professions education
RTA: reflexive thematic analysis
SANQF: South African National Qualifications Framework
SBL: simulation-based learning
VR: virtual reality
