# Supplementary material for: Virtual Reality in Health Professions Education: Qualitative Descriptive Study of Educators’ Perspectives
Source: JMIR XR Spat Comput. 2026 Jun 26;3:e52925. doi: 10.2196/52925 (PMC13308907; doi:10.2196/52925)
Supplement: Multimedia Appendix 1 [file xr-v3-e52925-s001.docx]

**Appendix 1: VR application details**

The Body VR: Journey Inside a Cell was selected to support foundational anatomical and physiological learning at the cellular level. Its guided, narrative-based format provides a conceptual overview of cellular structures and processes, aligning with introductory coursework that emphasises conceptual understanding rather than procedural skill development. The predominantly passive design was appropriate for illustrating abstract microscopic concepts that are difficult to visualise using traditional teaching approaches.

Related link:

<https://store.steampowered.com/app/451980/The_Body_VR_Journey_Inside_a_Cell/>

Sharecare You VR was chosen to support system-level anatomy and functional understanding of human organs. The application enables exploration of anatomical structures from multiple spatial perspectives and incorporates interactive elements and formative self-assessment via multiple-choice questions. This aligns with course requirements that emphasise spatial reasoning, integration of structure and function, and opportunities for self-directed learning and knowledge consolidation.

Related link: <https://steamcommunity.com/app/724590>

Wraith VR Total Knee Replacement Surgery Simulation was selected to represent a clinically oriented, procedural VR application relevant to musculoskeletal and rehabilitation-related curricula. The simulation exposes users to a realistic operating theatre environment and surgical workflow, supporting learning outcomes related to clinical exposure, procedural awareness, and professional context. Its higher level of interactivity and requirement for spatial navigation contrast with other applications and reflect more advanced clinical learning expectations.

Related link:

<https://store.steampowered.com/app/1136210/Ghost_Productions_Wraith_VR_Total_Knee_Replacement_Surgery_Simulation/>
