# Supplementary material for: Virtual Reality in Health Professions Education: Qualitative Descriptive Study of Educators’ Perspectives
Source: JMIR XR Spat Comput. 2026 Jun 26;3:e52925. doi: 10.2196/52925 (PMC13308907; doi:10.2196/52925)
Supplement: Multimedia Appendix 2 [file xr-v3-e52925-s002.docx]

**Appendix 2: Semi-structured Interview Guide**

Demographics and general VR questions:

1. Name:
2. Surname:
3. Which faculty are you teaching in?
4. What is your HPCSA profession?
5. Age: 20 - 29, 30 - 39, 40 - 49, 50 - 59, 60+
6. Are you familiar with Virtual Reality (VR)?
7. Have you ever used VR before?
   1. If yes, for what?
8. Would you be interested in attending workshops to create your own VR lessons?

Semi-structured interview:

1. How was your overall VR experience?
   1. If good:
      1. What do you like about it?
      2. What value do you see in it?
   2. If bad:
      1. What did you not enjoy?
   3. Other:
      1. Comfortable?
      2. Cybersickness?
      3. Immersive?
      4. Realistic?
2. How did you find the product or system (VR) difficult to use?
   1. If yes:
      1. What was difficult?
      2. What struggles did you face?
   2. If not:
      1. What made it easy to use?
3. Did you have any difficulty with the Head Mount Display?
   1. Yes
      1. What was it?
         1. The bulkiness?
         2. Blurry?
         3. Laggy images
         4. Too much inconsistency?
   2. No
      1. Was everything smooth and easy to use?
4. Do you think VR is something you can quickly learn to use?
   1. Yes:
      1. How long do you think it’ll take you to get used to setting up and using VR?
   2. Not:
      1. What is hindering the ease of using VR?
      2. What do you think you need before making it easy to use?
5. Did you have any difficulty with the VR hand controller?
   1. Yes
      1. What was it?
         1. Accuracy?
         2. Loss of controller tracking?
         3. Too much inconsistency?
   2. No
      1. Was everything smooth and easy to use?
      2. Zooming in/out
      3. Rotating
      4. Manipulating
6. The navigation through the structures menu has been intuitive?
   1. If yes:
      1. What made it that way?
   2. If not:
      1. How can it be improved?
7. Do you think you can set up this VR station on your own?
   1. If not,
      1. Are you interested in learning?
         1. If not, what would you like in place of it?
8. Do you think you will use this in your class?
   1. If yes:
      1. Which part of VR do you think is suitable for your classroom?
      2. What module will you implement them in (describe what the module is able to link to VR)?
   2. If not:
      1. Why will you not use it in your class?
      2. What do you need?
9. Do you think this will enhance your teaching?
   1. If yes:
      1. How?
   2. If not:
      1. Why not?
10. Do you think VR can change the way you teach?
    1. If yes:
       1. How?
    2. If not:
       1. Why not?
11. Do you think VR can change the way students learn?
    1. If yes:
       1. How?
    2. If not:
       1. Why not?
12. What do you think the advantages of VR education will be?
13. What do you think the disadvantages of VR education will be?
14. What are the key components you think are needed for a successful VR integration in the educational system?
