# Supplementary material for: Virtual Reality in Health Professions Education: Qualitative Descriptive Study of Educators’ Perspectives
Source: JMIR XR Spat Comput. 2026 Jun 26;3:e52925. doi: 10.2196/52925 (PMC13308907; doi:10.2196/52925)
Supplement: Multimedia Appendix 3 [file xr-v3-e52925-s003.docx]

**Appendix 3: Faculty of Community and Health Science**

*Details of the programme delivered at the University*

| **#** | **Department/School** | **Type of Degree** | **Years of study** | **Number of students (entire program)** |
| --- | --- | --- | --- | --- |
| 1 | Physiotherapy | Bachelor of Science (BSc) Honours in Physiotherapy | Four | ±240 |
| 2 | Occupational Therapy | Bachelor of Science (BSc) Honours in Occupational Therapy | Four | ±200 |
| 3 | Dentistry | Bachelor of Dental Surgery (BDS) | Five | ±300 |
| 4 | Nursing | Bachelor of Nursing (BN) | Four | ±650 |
| 5 | Dietetics and Nutrition | Bachelor of Science (BSc) in Dietetics | Four | ±160 |
| 6 | Psychology | Bachelor of Science (BSc) in Psychology | Four | ±450 |
| 7 | Social Work | Bachelor of Social Work (BSW) | Four | ±320 |
| 8 | Sports, Recreation and Exercise Science | Bachelor of Science in Sport, Recreation, and Exercise Science” (BSc SRES) | Three | ±400 |
| 9 | Biokineticist | Bachelor of Science (Honours) in Biokinetics | Four | ±240 |
| 10 | Interprofessional Education Units | Integrated into other Health Science modules and the Postgraduate Diploma | | |
| 11 | Complementary Health Science | Bachelor of Science in Complementary Health Sciences: Chinese Medicine and Acupuncture. | Five | ±100 |
| 12 | School of Public Health | Postgraduate Diploma, a Master’s, and a PhD | | |
| 13 | Community Development | Bachelor of Community Development | Four | ±200 |

*Table two: Participant details*

| **#** | **Department or School** | **Title** | **Speciality or area of teaching** | **Age** | **Health Professional Council Registration** |
| --- | --- | --- | --- | --- | --- |
| 1 | Physiotherapist | Lecturer | Clinical Coordinator and lecturer | 40 | HPCSA ^1^ |
| 2 | Physiotherapist | Lecturer | Clinical reasoning, ethics and Musculoskeletal | 33 | HPCSA |
| 3 | Physiotherapist | Associate Lecturer | Movement Science, Exercise Physiotherapy and Neurology | 38 | HPCSA |
| 4 | Psychology | Lecturer | Clinical Psychology | 32 | HPCSA |
| 5 | Psychology | Associate Lecturer | Brain and body psychology, and research psychology | 32 | HPCSA |
| 6 | Social Work | Lecturer | Basic social work, fieldwork, technology and advanced fieldwork. | 54 | SACSSP ^2^ |
| 7 | Sport Science | Associate Lecturer | Fundamentals of Exercise Physiology | 62 | HPCSA |
| 8 | Exercises and Rehabilitation Science | Senior Lecturer | Game concepts in Paralympic sports, basic Principles of Sport and Recreation Management. | 36 | N/A ^3^ |
| 9 | Sport Science | Professor | Fundamentals of Exercise Physiology, Sport in Society and Recreation | 40 | HPCSA |
| 10 | Exercises and Rehabilitation Science | Lecturer | Applied sport management  and advanced Exercise Physiology | 36 | N/A |
| 11 | Exercises and Rehabilitation Science | Lecturer | Sports Performance, Judgment and Decision Making, and Motor Control. | 32 | N/A |
| 12 | Physiotherapist | Lecturer^5^ | Health Promotion and Interprofessional Education. | 33 | HPCSA |
| 13 | Dentist | Senior Lecturer | Paediatric dentistry | 37 | HPCSA |
| 14 | Dentist | Senior Lecturer | Patient management, dental fears/phobias, Conservative Dentistry and Dental Education | 51 | HPCSA |
| 15 | Dentist | Senior Lecturer | Community Health and General Dentistry | 51 | HPCSA |
| 16 | Dentist | Lecturer | Paediatric Dentistry | 54 | HPCSA |
| 17 | Occupational Therapist | Lecturer | Orthopaedics and Public Health | 45 | HPCSA |
| 18 | Nursing | Lecturer | Child Health and Primary Health Care | 50 | SANC ^4^ |

*Key:*

1. *HPCSA: Health Professional Council of South Africa*
2. *SANC: South African Nursing Council*
3. *N/A: Not applicable (however, it is recommended that they register with associations like the Register of Exercise Professionals South Africa).*
4. *SACSSP: South African Council for Social Service Professions*
5. *Participant twelve is a physiotherapist by practice; however, they are currently employed by the IPEU. Therefore, this participant will be known as ‘PT’ in the results and discussion.*
