# Supplementary material for: Virtual Reality in Health Professions Education: Qualitative Descriptive Study of Educators’ Perspectives
Source: JMIR XR Spat Comput. 2026 Jun 26;3:e52925. doi: 10.2196/52925 (PMC13308907; doi:10.2196/52925)
Supplement: Multimedia Appendix 4 [file xr-v3-e52925-s004.docx]

**Appendix 4: Themes, Subthemes and Codes**

| **Themes** | **Subthemes** | **Codes** |
| --- | --- | --- |
| Theme 1: Experience of Virtual Reality | 1. Initial Impressions | - Prior awareness of VR terminology - VR associated with gaming - Absence of VR exposure in prior education - Curiosity before first use - Uncertainty before engagement - Surprise at VR capabilities - Novelty of immersive environment - Unexpected realism - Initial disorientation - Immediate emotional reaction - First-time “wow factor” - Rapid acclimatisation - Shift from scepticism to interest |
|  | 1. User Engagement | - Feeling immersed in the environment - Enjoyment during VR use - Fun as a motivator - Interactive exploration - Active manipulation of virtual objects - Sustained attention - Increased focus compared to lectures - Sense of presence - Feeling part of the learning environment - Multisensory engagement - Curiosity-driven exploration - Engagement through movement - Visual–spatial interaction - Learner agency within VR |
|  | 1. Learning Enhancement | - Enhanced spatial understanding - Seeing structures in context - Reduced the abstraction of anatomy with deeper understanding compared to textbooks - Improved conceptual clarity - Linking labels to structures - Understanding relationships between systems - Improved retention - Learning through visualisation - Integration of audio and visual cues - Reinforcement through exploration - Meaningful learning experience |
| Theme 2: Teaching and Learning Preferences | - 1. Explorative Learning | - Freedom to explore content with discovery-based learning - Learner-directed navigation - Choice-driven pathways - Revisiting areas of interest - Investigating from different angles - Learning through exploration - Trial-and-error exploration - Curiosity-led engagement |
|  | - 1. Individualised Learning | - Self-paced and support slower learning - Ability to repeat sections - Customised learning speed - Adaptation to learner needs - Autonomous control of learning - Personalised learning pathways - Reduced pressure compared to tutorials - Independent exploration - Learning without peer comparison |
|  | - 1. Authentic Learning | - Realistic simulation of environments - Clinical realism - “Almost-real” scenarios - Simulated patient interactions - Contextual learning - Application to real-world practice - Learning in situational contexts - Bridging theory and practice - Safe approximation of clinical settings - Preparation for real clinical encounters |
| Theme 3: Challenges of VR | 1. Visual Overload | - Excessive visual stimulation - Cognitive overload (too much information at once) - Difficulty processing multiple cues - Overwhelming sensory input - Distraction from learning objectives - Reduced focus due to rich visuals - Need for scaffolded design - Visual fatigue - Initial overload during first exposure |
|  | 1. Cybersickness | - Loss of balance - Physical discomfort - Motion sickness symptoms - Blurred vision - Dizziness - Physiological strain - Fatigue during use - Adjustment difficulties - Short tolerance duration |
|  | 1. Ease of Use | - Difficulty using controllers (including button usage) - Complex button layout - Lack of intuitive controls - Learning curve for device operation - Technical glitches - Lag in system response - Need for user training - Fine motor control challenges - Interface usability issues - Time required to become a competent user |
|  | 1. Cost and Accessibility | - High initial investment cost - Limited institutional budgets - Need for multiple headsets - Large class sizes - Equipment sharing constraints - Time inefficiency for rotations - Infrastructure limitations - Equity of student access - Cost-effectiveness concerns - Scalability challenges |
| Theme 4: Clinical Competencies and Student Confidence | - 1. Pre-Clinical Training | - Preparation and familiarisation before clinical placement - Skill rehearsal - Early exposure to procedures to reduce anxiety - Bridging pre-clinical and clinical phases - Practising without patient presence - Repetition before real-world exposure |
|  | - 1. Clinical Simulations | - Simulated patient scenarios - Exposure to varied clinical cases - Practice of assessment skills - Treatment planning in VR - Managing complex conditions - Experiencing rare cases - Safe practice of procedures - Clinical decision-making rehearsal |
|  | - 1. Patient Safety | - Learning without risk to patients - Error-making without harm - Safe environment for mistakes - Ethical protection of patients - Reducing clinical errors - Confidence before patient contact - Risk mitigation - “No harm” principle - Non-threatening learning environment |
| Theme 5: Integration of VR into Curriculum | - 1. Year Level Suitability | - Alignment with student seniority - Junior-year anatomy focus - Progressive skill complexity - Scaffolding across years - Foundation building in early years - Advanced applications for senior students - Postgraduate suitability - Developmental appropriateness |
|  | - 1. Module Alignment | - Alignment with module outcomes - Curriculum coherence - Learning outcome-driven design - Narrow and specificity of Professional focus - Avoiding generic VR use - Constructive alignment - Curriculum integration planning |
|  | - 1. Teaching Methodology | - VR as primary teaching tool - VR as a supplementary tool - Replacement of lectures - Blended learning approaches - Pre-class preparation - Flipped classroom integration - Practice-oriented teaching - Balance between theory and application - Pedagogical flexibility |
